# Paired In-Hospital Dynamics in Hepatitis E: Rapid Transaminase Decline and Persistent Hyperbilirubinemia in a Romanian Cohort

**DOI:** 10.3390/diagnostics16071012

**Published:** 2026-03-27

**Authors:** Florentina Dumitrescu, Eugenia-Andreea Marcu, Vlad Pădureanu, Virginia Maria Rădulescu, Ion Rogoveanu

**Affiliations:** 1 Department of Infectious Disease, University of Medicine and Pharmacy of Craiova, 200349 Craiova, Romania; 2 ”Victor Babes”, Hospital of Infectious Diseases and Pulmonology from Craiova, 200515 Craiova, Romania; 3 Department of Internal Medicine, University of Medicine and Pharmacy of Craiova, 200349 Craiova, Romania; 4 Department of Medical Informatics and Biostatistics, University of Medicine and Pharmacy of Craiova, 200349 Craiova, Romania; 5 Department of Gastroenterology, University of Medicine and Pharmacy of Craiova, 200349 Craiova, Romania

**Keywords:** hepatitis E, acute hepatitis, hyperbilirubinemia, transaminases, cholestasis, sex differences, hospitalization, Romania, viral hepatitis, real-world cohort

## Abstract

**Background/Objectives**: Hepatitis E virus (HEV) infection is an increasingly recognized cause of acute hepatitis in Europe, but short-term in-hospital laboratory dynamics remain insufficiently described in hospitalized cohorts. We aimed to characterize admission biochemical abnormalities and paired admission-to-discharge laboratory changes in hospitalized patients with acute hepatitis E from Craiova, Romania, with exploratory sex- and age-stratified analyses. **Methods**: We conducted a single-center retrospective observational study including 40 consecutive hospitalized patients with acute hepatitis E during 2024–2025. Admission and discharge laboratory values were compared at the within-patient level, and exploratory subgroup analyses by sex and age class were performed. Given the limited sample size, multivariable analyses were restricted to parsimonious age-adjusted models for selected endpoints. **Results**: The cohort comprised 22 females (55%) and 18 males (45%), with a mean age of 53.05 ± 21.44 years; two in-hospital deaths occurred. At admission, marked transaminase elevation and frequent hyperbilirubinemia were observed, with 70% of patients having total bilirubin ≥ 2 mg/dL and 40% ≥ 10 mg/dL. During hospitalization, ALT and AST declined markedly, whereas total and direct bilirubin improved more modestly, indicating slower resolution of jaundice/cholestatic abnormalities. Platelets increased, while prothrombin index changes were heterogeneous. Male patients had higher bilirubin values at admission and discharge and more frequent clinically relevant hyperbilirubinemia thresholds; however, these findings should be interpreted cautiously given the small sample size, the retrospective design, and the absence of standardized clinical confounders and mechanistic data. Exploratory age-stratified analyses did not identify robust differences after multiplicity control. **Conclusions**: In hospitalized hepatitis E, hepatocellular injury markers improved rapidly during hospitalization, whereas cholestatic abnormalities resolved more slowly and often remained clinically relevant at discharge. The observed sex-related cholestatic pattern should be considered exploratory and requires confirmation in larger studies with standardized clinical covariates and longer follow-up. These findings support closer monitoring of bilirubin trajectories at discharge, particularly in male patients, and highlight the need for integrating laboratory dynamics into short-term clinical assessment of hospitalized HEV cases.

## 1. Introduction

Hepatitis E virus (HEV) is an important cause of acute viral hepatitis worldwide and represents an emerging public health concern due to its wide geographic distribution, genetic diversity, and complex transmission pathways. Once considered a disease confined mainly to regions with poor sanitation and limited healthcare infrastructure, HEV infection is now increasingly recognized in industrialized countries, where zoonotic transmission plays a major role. The growing number of reported cases, together with improved diagnostic capabilities, has contributed to a renewed interest in the virology, epidemiology, and clinical impact of HEV infection [1,2].

HEV belongs to the Hepeviridae family and is a small, non-enveloped virus containing a positive-sense, single-stranded RNA genome. The virus displays considerable genetic variability, which has been shown to influence host specificity, transmission routes, pathogenicity, and geographic distribution [3].

Based on phylogenetic analysis, HEV strains are classified into several genotypes, of which at least four (genotypes 1–4) are known to infect humans. Genotypes 1 and 2 are restricted to humans and are predominantly associated with large waterborne outbreaks in developing countries. In contrast, genotypes 3 and 4 are zoonotic, infecting a wide range of animal species—particularly domestic pigs, wild boar, and deer—and are responsible for sporadic and autochthonous infections in both developing and industrialized regions [1,3]. Additional HEV genotypes have been identified in animals such as camels, rabbits, and rodents, raising concerns about their potential zoonotic transmission and implications for human health [1].

The primary mode of HEV transmission is the fecal–oral route, although the relative importance of specific transmission pathways varies according to genotype and geographic context. In regions where genotypes 1 and 2 are endemic, HEV infection is most commonly associated with the consumption of contaminated drinking water, leading to large outbreaks affecting thousands of individuals. Such outbreaks are frequently observed in settings characterized by inadequate sanitation, poor water quality, and overcrowding, particularly in parts of Asia, Africa, and Latin America [1].

In contrast, in non-endemic regions, zoonotic transmission is considered the predominant route of infection. HEV genotypes 3 and 4 are transmitted mainly through the consumption of undercooked or raw meat products, especially pork and game meat. Several studies have demonstrated a high prevalence of HEV RNA and antibodies in swine populations, supporting the role of pigs as a major reservoir for human infection. Occupational exposure, such as among farmers, veterinarians, and slaughterhouse workers, has also been associated with an increased risk of HEV seropositivity [1,3].

Beyond foodborne transmission, additional routes of HEV transmission have gained increasing attention in recent years. Transfusion-transmitted HEV infection has been documented in multiple countries, leading to the implementation of blood donor screening programs in some regions. Vertical transmission from mother to fetus has been reported, particularly in infections caused by genotype 1, and is associated with severe outcomes, including fulminant hepatitis and increased maternal and fetal mortality [4].

The World Health Organization estimates that approximately 20 million HEV infections occur annually worldwide, resulting in several million symptomatic cases and tens of thousands of deaths each year . However, the true incidence is likely underestimated due to limited surveillance, underdiagnosis, and the high proportion of asymptomatic or mildly symptomatic infections.

The geographic distribution of HEV genotypes shows marked regional variation. Genotypes 1 and 2 remain endemic in large parts of South and Southeast Asia, sub-Saharan Africa, and Latin America, where periodic outbreaks continue to occur [5,6]. These genotypes disproportionately affect young adults and are associated with particularly severe disease in pregnant women, especially during the third trimester. In contrast, genotypes 3 and 4 exhibit a broader geographic distribution, including Europe, North America, East Asia, and parts of Africa, where infections are mainly sporadic and often occur in older individuals or those with underlying comorbidities [1,7].

In recent years, HEV infection has increasingly emerged as a significant public health concern across Europe, with a notable rise in reported cases and measurable seroprevalence among the general population. According to surveillance data from the European Centre for Disease Prevention and Control (ECDC), the number of confirmed HEV cases in EU/EEA countries increased approximately ten-fold from 514 cases in 2005 to 5617 cases in 2015, reflecting both increased awareness and improved diagnostic capacity for HEV infection in the region. During this period, more than 21,000 acute clinical cases were reported, along with at least 28 associated deaths, although differences in national surveillance systems make direct comparison between countries difficult [8,9].

Serological studies demonstrate substantial variation in HEV seroprevalence across Europe. A systematic review reported that the pooled anti-HEV IgG positivity rate in the European population was approximately 9.31%, while anti-HEV IgM seroprevalence was around 0.79%, and HEV RNA positivity was around 0.08%, underscoring the presence of both prior exposure and active infection in some individuals. These figures position Europe above North America and Oceania in terms of seroprevalence, yet lower than in Africa and Asia, where HEV exposure rates are generally higher [10,11].

In summary, HEV is a genetically diverse virus with multiple transmission routes and a complex global epidemiology. The evolving patterns of HEV infection—characterized by the persistence of waterborne outbreaks in endemic regions and the increasing recognition of zoonotic, foodborne transmission in industrialized countries, including Europe—highlight the dynamic nature of this infection [1]. The substantial seroprevalence observed in the general population, together with the rising number of reported cases, underscores the public health relevance of HEV, particularly for vulnerable groups such as pregnant women, immunocompromised individuals, and patients with underlying liver disease [4,9,11]. A comprehensive understanding of HEV virology, transmission pathways, and regional epidemiology is therefore essential to improve diagnostic accuracy, guide clinical management, and inform effective surveillance and prevention strategies worldwide [1,8].

Although hepatitis E is usually a self-limiting acute viral hepatitis, its clinical presentation is not always limited to a transient hepatocellular injury pattern. In some patients, HEV infection may be accompanied by marked jaundice and a more prolonged cholestatic evolution, with bilirubin abnormalities remaining clinically relevant even after aminotransferases begin to decline. This distinction is particularly important in hospitalized patients, in whom early biochemical recovery of hepatocellular injury may coexist with persistent icteric burden.

## 2. Materials and Methods

### 2.1. Study Design and Setting

This retrospective observational study included consecutive hospitalized patients diagnosed with hepatitis E and managed in a hospital department/ward in Craiova, Romania. The study period covered January 2024 to December 2025. This interval was selected to capture the most recent consecutive cases of acute hepatitis E diagnosed and managed in our center within a uniform contemporary clinical and laboratory framework. Clinical and laboratory data were extracted from routinely collected hospital records and organized in a structured dataset. Hospitalization reflected routine clinical practice for patients with acute hepatitis and generally involved clinically significant jaundice, marked liver enzyme abnormalities, systemic symptoms, diagnostic uncertainty, or the need for in-hospital monitoring and supportive care. The study aimed to describe baseline clinical-laboratory features at admission and to quantify within-hospital dynamics from admission to discharge, with exploratory sex- and age-stratified analyses and parsimonious age-adjusted models.

### 2.2. Study Population and Operational Definitions of Variables and Outcomes

#### 2.2.1. Eligibility Criteria

Eligible patients were consecutive hospital admissions during 2024–2025 with a diagnosis of acute hepatitis E established by the treating team based on anti-HEV IgM positivity, a compatible acute hepatitis clinical–biological syndrome, and exclusion of alternative causes of acute hepatocellular injury through history, laboratory testing, and imaging assessment. *Inclusion criteria*:

- Hospitalization during 2024–2025 with hepatitis E diagnosis.
- Availability of core demographic variables (age, sex, year).
- Availability of admission laboratory measurements for the primary endpoints.

*Exclusion criteria*:

Other causes of hepatocytolysis syndrome include:

- Hepatitis viruses: HAV, HBV, HCV, HDV;
- Other viruses: EBV (Epstein–Barr), CMV (cytomegalovirus), HSV (herpes simplex virus);
- Alcohol consumption: Alcoholic hepatic steatosis, Alcoholic hepatitis, Alcoholic cirrhosis;
- Drugs and toxins (hepatotoxicity): Paracetamol (in overdose), Antituberculous drugs (isoniazid, rifampicin), Antiepileptic drugs, Antibiotics, Toxic mushrooms (e.g., Amanita phalloides), Industrial solvents;
- Metabolic diseases: MASLD, Hemochromatosis, Wilson’s disease, Alpha-1 antitrypsin deficiency;
- Autoimmune diseases: Autoimmune hepatitis, Primary biliary cholangitis, Primary sclerosing cholangitis;
- Ischemic causes: Shock, Severe heart failure, Prolonged hypotension;
- Other causes: Liver trauma, Liver tumor infiltration, Sepsis, Rhabdomyolysis.

All these possible etiologies for hepatocytolysis syndrome were ruled out through history, laboratory, and imaging investigations.

#### 2.2.2. Data Collection and Preprocessing

Data were collected, curated, and cleaned in Microsoft Excel 365 (Microsoft Corporation, Redmond, WA, USA), including harmonization of variable names and removal of empty spaces. For laboratory variables, suffixes were used to denote time points: “_int” for admission and “_ext” for discharge.

#### 2.2.3. Variables and Definitions

Demographics:

Year of hospitalization: 2024 vs. 2025;

Sex: female vs. male (as recorded);

Age: years at admission (continuous);

Age classes (exploratory);

For stratified analyses, age was categorized as ≤55 years vs. >55 years, selected to provide reasonably balanced groups in this small cohort.

*Laboratory* *markers (recorded at admission and discharge)*

ALT (GPT), U/L;

AST (GOT), U/L;

Total bilirubin (TB), mg/dL;

Direct bilirubin (DB), mg/dL;

Alkaline phosphatase (ALP), U/L;

Gamma-glutamyl transferase (GGT), U/L;

Prothrombin index (IP), %;

Leukocytes, /µL;

Platelets, /µL.

*Derived variable*

De Ritis ratio: AST/ALT, computed at both time points;

Clinically relevant thresholds (descriptive + inferential where specified);

Total bilirubin: TB ≥ 2 mg/dL, TB ≥ 5 mg/dL, TB ≥ 10 mg/dL;

Coagulation impairment (descriptive): IP < 60%.

Within-hospital change variables

For each marker: Δ = discharge (_ext) − admission (_int).

Although liver injury patterns can be classified using the R-ratio according to established guidelines, this categorization was not used as a primary analytical framework in the present study, which focused on within-patient laboratory dynamics rather than baseline phenotypic classification.

*Pattern of liver injury*

To align the analysis with established clinical frameworks, the pattern of liver injury was defined using the R-ratio, calculated as (ALT/ULN ALT)/(ALP/ULN ALP). Based on this ratio, liver injury was categorized as hepatocellular (R ≥ 5), cholestatic (R ≤ 2), or mixed (R between 2 and 5), in accordance with validated clinical guidelines for the evaluation of abnormal liver chemistries [12].

#### 2.2.4. Outcomes

Primary outcome: within-patient change from admission to discharge in hepatocellular injury markers (ALT, AST).

Secondary outcomes: within-patient changes in TB, DB, De Ritis ratio, platelets, leukocytes, and IP;

Sex-stratified differences at admission and discharge (continuous markers and threshold-based endpoints);

Exploratory age-class contrasts (≤55 vs. >55 years) for admission/discharge markers;

Exploratory outcomes: Spearman correlation patterns among admission markers;

Parsimonious age-adjusted models assessing the association of sex with bilirubin fractions and leukocytes (log-linear), and discharge IP (linear), including baseline-adjusted discharge models.

### 2.3. Statistical Analysis

Data preprocessing and variable derivation were undertaken in Microsoft Excel 365, and statistical analyses were performed using IBM SPSS Statistics for Windows, version 26.0 (IBM Corp., Armonk, NY, USA). All statistical tests were two-sided with a significance level of α = 0.05.

Continuous variables were summarized using median and interquartile range (IQR), with minimum and maximum values reported to reflect the distribution in this small clinical cohort; mean ± standard deviation was additionally reported for age. Categorical variables were summarized as counts and percentages.

Within-patient admission-to-discharge changes were evaluated using the Wilcoxon signed-rank test. Differences between sexes and between exploratory age classes (≤55 vs. >55 years) were assessed using the Mann–Whitney U test for continuous variables and Fisher’s exact test for categorical endpoints. Associations among admission laboratory markers were explored using Spearman’s rank correlation.

Given the limited sample size, multivariable analyses were restricted to parsimonious models including only key covariates. Log-linear regression models were applied for right-skewed outcomes (bilirubin fractions and leukocytes), and linear regression models were used for discharge prothrombin index. Selected models additionally adjusted for baseline values to evaluate whether discharge differences persisted beyond admission severity.

*To reduce the risk of false-positive findings across multiple comparisons, false discovery rate correction (Benjamini–Hochberg method) was applied within predefined families of related tests.*

### 2.4. Ethics

The study received approval from the Ethics Committee of the University of Medicine and Pharmacy of Craiova (approval number 22/03.02.2026) and was conducted in accordance with the Declaration of Helsinki. Informed consent was obtained from all subjects involved in the study.

## 3. Results

### 3.1. Study Population and Baseline Presentation

Between 2024 and 2025, 40 hospitalized patients with hepatitis E were included from a hospital department in Craiova, Romania (2024: *n* = 20; 2025: *n* = 20). The sex distribution did not differ by year (2024: 12 females/8 males; 2025: 10 females/10 males; Fisher’s exact *p* = 0.751). Age was 57.5 years (IQR 38.8–63.0; range 16–85; mean 53.05 ± 21.44), with evidence against normality (Shapiro–Wilk *p* = 0.0066). For exploratory age-stratified analyses, patients were grouped as ≤55 years (*n* = 18) versus >55 years (*n* = 22). The distribution across these age classes was relatively balanced, supporting their use for exploratory comparisons in this small cohort. Overall, the cohort comprised 22 females (55%) and 18 males (45%), with two in-hospital deaths (5.0%, occurred during the study period). Because the present study focused on admission-to-discharge laboratory dynamics rather than on clinical outcome modeling, detailed analyses of mortality predictors were beyond the scope of this dataset. At admission, laboratory findings were consistent with acute hepatitis, with marked elevations in transaminases and frequent hyperbilirubinemia (Table 1). When examined by sex, male patients tended to present higher bilirubin values and a greater proportion of clinically relevant hyperbilirubinemia thresholds, suggesting a more pronounced icteric/cholestatic component at baseline. Overall, 28/40 (70%) had total bilirubin ≥ 2 mg/dL, 18/40 (45%) had total bilirubin ≥ 5 mg/dL, and 16/40 (40%) had marked hyperbilirubinemia (total bilirubin ≥10 mg/dL). The De Ritis ratio was <1 in 30/40 (75%), suggesting a predominantly ALT-driven hepatocellular pattern, while coagulation impairment (prothrombin index < 60%) was present in 16/40 (40%). The laboratory profile was consistent with a predominantly hepatocellular pattern of injury at presentation, based on De Ritis ratio distribution. Both in-hospital deaths occurred in elderly males and coincided with extreme hyperbilirubinemia at presentation.

### 3.2. Within-Hospital Dynamics from Admission to Discharge

#### 3.2.1. Hepatocellular Injury Markers (ALT/AST)

**Figure 1:**
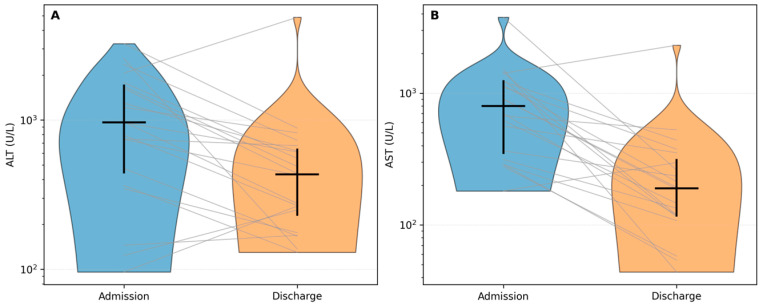
Hepatocellular injury markers during hospitalization: (**A**) ALT (GPT) at admission and discharge; (**B**) AST (GOT) at admission and discharge. Blue and orange violins represent the admission and discharge distributions, respectively. Gray lines connect paired measurements from the same patient. Black markers indicate the cohort median (horizontal tick) and interquartile range (IQR; vertical bar). Log scale.

Across hospitalization, transaminases showed a pronounced and consistent decline, indicating improvement in hepatocellular injury. ALT and AST decreased significantly from admission to discharge (both Wilcoxon *p* < 0.001; BH-FDR *q* < 0.001; Table 2). Patient-level paired trajectories are shown in Figure 1A,B (log scale), highlighting both the overall downtrend and the inter-individual variability typical of acute viral hepatitis presentations.

At the individual level, ALT decreased in 32/40 patients (80%), and AST decreased in 36/40 (90%). The median relative change was −58% for ALT and −78% for AST, consistent with biochemical recovery during hospitalization (Table 2; Figure 1A,B).

#### 3.2.2. Jaundice/Cholestasis Markers

**Figure 2:**
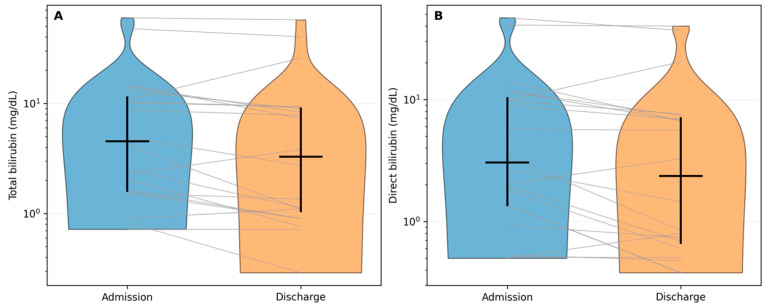
Jaundice/cholestasis markers during hospitalization. (**A**) Total bilirubin (BT) at admission and discharge. (**B**) Direct bilirubin (BD) at admission and discharge. Blue and orange violins represent the admission and discharge distributions, respectively. Gray lines connect paired measurements from the same patient. Black markers indicate the cohort median and IQR. Log scale.

Markers of jaundice/cholestasis also improved during hospitalization. Total bilirubin declined from a median of 4.53 mg/dL to 3.27 mg/dL and direct bilirubin from 3.04 mg/dL to 2.36 mg/dL (both Wilcoxon *p* < 0.001; BH-FDR *q* < 0.001; Table 2), with paired trajectories shown in Figure 2A,B (log scale). At the individual level, total bilirubin decreased in 32/40 patients (80%), increased in 6/40 (15%), and was unchanged in 2/40 (5%); direct bilirubin showed the same pattern (32/40 decreased, 6/40 increased, 2/40 unchanged). The median relative change was approximately −24% for total bilirubin and −22% for direct bilirubin. The De Ritis ratio decreased in parallel (*p* < 0.001; *q* < 0.001), consistent with a recovery pattern in acute hepatitis E in most hospitalized cases.

### 3.3. Hematology and Synthetic Function Markers

**Figure 3:**
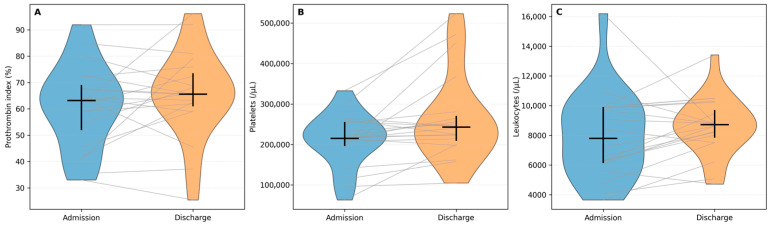
Selected hematology and liver function markers during hospitalization. (**A**) Prothrombin index (IP) at admission and discharge. (**B**) Platelet count (Tromb) at admission and discharge. (**C**) Leukocyte count (Leuc) at admission and discharge. Blue and orange violins represent the admission and discharge distributions, respectively. Gray lines connect paired measurements from the same patient. Black markers indicate the cohort median and IQR.

Platelet counts increased significantly from 215,000/µL to 243,000/µL (Wilcoxon *p* < 0.001; BH-FDR *q* < 0.001; Table 2), with increases observed in 32/40 patients (80%) (Figure 3B). In contrast, the prothrombin index (IP) did not show a consistent within-hospital change (*p* = 0.175; Table 2): IP increased in 20/40 patients, decreased in 18/40, and was unchanged in 2/40, indicating heterogeneous dynamics of synthetic function over the hospitalization interval (Figure 3A). Leukocyte counts showed a modest overall increase (*p* = 0.010; *q* = 0.011), rising in 28/40 patients (70%) and decreasing in 12/40 (30%) (Figure 3C), consistent with variable inflammatory activity and potential concomitant conditions in hospitalized patients.

### 3.4. Sex-Stratified Analyses

At admission, males showed a distinctly more cholestatic/icteric biochemical profile than females. Specifically, total bilirubin and direct bilirubin were substantially higher in males (both BH-FDR *q* ≤ 0.001; Table 3), alongside higher leukocyte counts (*q* ≤ 0.001; Table 3). In contrast, transaminases (ALT/AST), cholestatic enzymes (ALP, GGT), prothrombin index, and platelet counts did not show robust sex differences after multiplicity correction (all *q* ≥ 0.10; Table 3). Importantly, males were older than females (mean 65.11 ± 15.41 vs. 43.18 ± 20.85 years; Welch’s t-test *p* < 0.001), which may confound unadjusted sex contrasts and should be considered when interpreting these exploratory comparisons.

At discharge, the sex divergence in bilirubin persisted. Males continued to have markedly higher total and direct bilirubin than females (both *q* ≤ 0.002; Table 4), indicating more frequent residual cholestasis at the end of hospitalization in the male subgroup. In addition, leukocyte counts remained higher in males (*q* = 0.002; Table 4), while females had higher prothrombin index values at discharge (*q* = 0.002; Table 4), consistent with a more favorable recovery in synthetic function among females within this cohort. No statistically robust sex differences were observed for ALT, AST, or platelet counts at discharge after FDR adjustment (Table 4).

Threshold-based analyses supported the continuous-marker findings. At admission, clinically relevant hyperbilirubinemia was more frequent in males, particularly for total bilirubin ≥5 mg/dL (77.8% vs. 18.2%; Fisher’s exact *p* < 0.001) and ≥10 mg/dL (66.7% vs. 18.2%; *p* = 0.003) (Table 5). At discharge, persistent hyperbilirubinemia remained common in males: total bilirubin ≥2 mg/dL was present in 88.9% of males versus 27.3% of females (*p* < 0.001), and total bilirubin ≥5 mg/dL in 77.8% versus 18.2% (*p* < 0.001) (Table 5).

When comparing within-hospital changes (Δ discharge − admission), several sex differences remained detectable after FDR correction (Table 6). Females exhibited larger ALT reductions (median Δ −675 vs. −234 U/L; BH-FDR *q* = 0.040) and larger platelet increases (median Δ +44,000 vs. +9000/µL; *q* = 0.040), whereas males showed larger absolute decreases in total bilirubin (median Δ −2.61 vs. −0.73 mg/dL; *q* = 0.040). Differences in AST change did not remain statistically robust after multiplicity correction (*q* = 0.070), and no sex differences were observed for changes in direct bilirubin, leukocytes, prothrombin index, or De Ritis ratio after FDR adjustment (Table 6). Overall, these sex-stratified patterns suggest that male patients tended to present with (and partially retain) a stronger cholestatic phenotype, while females showed comparatively more favorable in-hospital dynamics for hepatocellular injury (ALT) and platelet recovery; given the older age distribution in males, age-adjusted modeling is warranted and is addressed in the subsequent multivariable analyses.

### 3.5. Age-Stratified Differences (≤55 vs. >55 Years)

In exploratory analyses stratified by age (≤55 years, *n* = 18 vs. >55 years, *n* = 22), no admission or discharge laboratory marker showed a statistically robust between-group difference after BH-FDR adjustment (Table 7). At admission, the ≤55 group displayed a nominally lower prothrombin index compared with the >55 group (median 61.53% vs. 64.21%; *p* = 0.035), but this contrast did not remain significant after multiplicity correction (*q* = 0.242). At discharge, ALT levels were higher in the ≤55 group (median 612 vs. 267 U/L; *p* = 0.015), yet this also did not persist after BH-FDR correction (*q* = 0.208). All other admission and discharge markers (AST, bilirubin fractions, leukocytes, and platelets) were broadly comparable across age strata (all *q* ≥ 0.491), suggesting that age-class stratification did not materially refine baseline severity or short-term in-hospital biochemical evolution beyond the sex-based contrasts observed in this cohort.

In complementary analyses focusing on within-hospital change (Δ discharge − admission) and clinically relevant hyperbilirubinemia thresholds by age class, we similarly observed no statistically robust differences between patients ≤55 versus >55 years. Overall, age-class stratification did not meaningfully refine risk or recovery patterns beyond the sex-based contrasts in this cohort.

### 3.6. Multivariable Analyses (Sex, Age-Adjusted)

In age-adjusted log-linear models, male sex was independently associated with higher bilirubin levels at both admission and discharge (Table 8). At admission, males had 4.65-fold higher total bilirubin (95% CI 1.53–14.09; *p* = 0.015) and 4.49-fold higher direct bilirubin (95% CI 1.29–15.58; *p* = 0.030); leukocyte counts were also higher in males (1.55-fold; 95% CI 1.12–2.14; *p* = 0.017). Age (modeled per 10-year increase) was not independently associated with these markers in the same models (all *p* ≥ 0.586).

At discharge, the sex-associated separation persisted, with males showing 7.10-fold higher total bilirubin (95% CI 2.07–24.40; *p* = 0.006) and 8.54-fold higher direct bilirubin (95% CI 2.38–30.65; *p* = 0.004) compared with females (Table 8). When discharge bilirubin was additionally adjusted for baseline (admission) bilirubin, the sex effect attenuated for total bilirubin (1.53-fold; *p* = 0.229) but remained statistically significant for direct bilirubin (2.16-fold; 95% CI 1.08–4.29; *p* = 0.044), suggesting that admission severity largely explains the discharge difference in total bilirubin, with a residual sex-associated signal for conjugated bilirubin (Table 8).

In a separate linear model of discharge prothrombin index (expressed in percentage points), male sex was associated with a lower discharge prothrombin index (−20.75 pp; 95% CI −36.85 to −4.64; *p* = 0.015), whereas adjustment for baseline prothrombin index attenuated this association (*p* = 0.089) (Table 9).

## 4. Discussion

Hepatitis E virus (HEV) infection has emerged as a major cause of acute viral hepatitis worldwide, with marked epidemiological contrasts between low- and high-income settings [12,13,14]. In Europe, HEV is increasingly recognized as an autochthonous infection, largely driven by zoonotic transmission and genotype 3 circulation [14,15]. Romanian data remain scarce, with only a limited number of studies documenting sporadic cases and low testing rates among patients presenting with acute hepatitis [15,16,17]. Against this background, our findings support an indigenous pattern of infection and reinforce the relevance of HEV as an underdiagnosed etiology in this region.

The observed epidemiological profile in our cohort is consistent with patterns reported across EU/EEA countries, where most cases are locally acquired and associated with zoonotic exposure rather than travel-related infection [14]. The absence of outbreak-type clustering, the predominance of adult and older patients, and the lack of recent travel history further support this interpretation. In addition, the documented circulation of HEV genotype 3 in the Romanian swine population [18], together with its known predominance among middle-aged and elderly individuals [19], suggests that the cases analyzed in this study are most likely linked to genotype 3 infection.

From a biochemical perspective, our results highlight a consistent and clinically relevant pattern: markers of hepatocellular injury declined rapidly between admission and discharge, whereas bilirubin fractions improved more modestly, and clinically significant hyperbilirubinemia frequently persisted at discharge. This dissociation suggests that, in hospitalized HEV infection, biochemical recovery of hepatocellular injury may precede the resolution of jaundice and cholestatic abnormalities.

Additional laboratory changes—including a decrease in the De Ritis ratio and an increase in platelet count—were observed during hospitalization. While these findings may reflect recovery from hepatocellular injury and systemic inflammatory stress, their interpretation remains non-specific. Overall, the paired laboratory dynamics point toward a temporal dissociation between rapid transaminase normalization and slower cholestatic improvement.

This discordant recovery pattern has been described in acute hepatitis, where cholestatic manifestations may persist despite improvement in hepatocellular injury, likely reflecting transient impairment of bile formation and hepatocellular transport mechanisms. In clinical practice, this may translate into a scenario in which patients exhibit biochemical and symptomatic improvement, yet remain visibly icteric at discharge.

Sex-related differences were also observed in our cohort, with male patients presenting higher bilirubin levels and a greater frequency of clinically relevant hyperbilirubinemia thresholds. However, these findings should be interpreted cautiously. The relatively small subgroup sizes, the retrospective design, and the lack of systematically recorded confounders—such as alcohol use, metabolic status, or viral genotype—limit causal inference. Consequently, these observations should be regarded as exploratory and hypothesis-generating, requiring confirmation in larger, well-characterized cohorts.

From a clinical standpoint, two practical implications emerge. First, the rapid decline in ALT and AST supports the notion that hepatocellular injury in acute HEV is often self-limited under supportive care. Second, the slower resolution of bilirubin highlights the need for careful clinical interpretation at discharge, as biochemical improvement does not necessarily equate to complete resolution of cholestatic manifestations. Importantly, due to the absence of standardized clinical severity indicators—such as ICU requirement, duration of jaundice, or post-discharge evolution—the present study can only infer clinical significance indirectly from laboratory trajectories rather than establish formal clinicobiological correlations.

Host-related factors may further modulate disease expression. Emerging evidence suggests that genetic variants affecting hepatobiliary transport and bile acid regulation—including ATP8B1, ABCB4, ABCB11, MYO5B, and the farnesoid X receptor pathway (NR1H4/FXR)—may predispose to prolonged cholestatic phenotypes. Although such mechanisms are not specific to HEV infection, they may contribute to interindividual variability in disease severity and recovery. In the present study, genetic testing was not available; therefore, this hypothesis remains speculative.

Marked hyperbilirubinemia in acute hepatitis E may, in some cases, mimic more severe hepatobiliary disorders and complicate the initial diagnostic approach. Although extreme bilirubin elevations are not the most typical presentation, they have been reported and should prompt consideration of HEV in the differential diagnosis of acute hepatitis, particularly in settings where other causes of severe liver injury are suspected.

Diagnostic challenges remain an important consideration. Variability in the sensitivity and specificity of commercially available anti-HEV assays has been documented [20,21,22], potentially affecting diagnostic accuracy. Current diagnostic approaches rely on the detection of anti-HEV IgM antibodies in combination with seroconversion or rising IgG titers, or on the identification of HEV-RNA in serum or feces. In Romania, IgG testing has been used predominantly in seroprevalence studies [15,16,23], while HEV-RNA detection remains limited by technical availability and a narrow diagnostic window during the acute phase [24,25].

Management of the cholestatic component in acute HEV infection is primarily supportive. In the broader context of cholestatic liver diseases, pharmacological agents such as rifampicin and bezafibrate have been explored; however, these approaches were not evaluated in the present study and should be interpreted as literature-based considerations rather than therapeutic implications derived from our data.

Two in-hospital deaths were recorded; however, the study was not designed to assess mortality predictors or detailed clinical trajectories. Given the limited sample size and the lack of standardized severity indicators, no robust conclusions regarding outcome determinants can be drawn. Future studies incorporating larger cohorts and structured clinical data will be necessary to better characterize prognostic factors in hospitalized HEV infection.

### Limitations of the Study

This study has several limitations that warrant consideration. The relatively small cohort size (*n* = 40), reflecting a single-center experience, limits statistical power and may obscure modest associations, particularly in subgroup analyses. In addition, the short admission-to-discharge observation window precludes evaluation of longer-term outcomes, including persistence of cholestasis, duration of jaundice, or delayed complications.

Furthermore, clinically relevant outcome variables were not systematically standardized in the retrospective dataset, preventing formal correlation between laboratory trajectories and endpoints such as ICU admission, hepatic decompensation, or post-discharge evolution. As such, the findings should be interpreted primarily as descriptive of short-term in-hospital biochemical dynamics.

Serological limitations also apply. The absence of quantitative anti-HEV IgG testing precluded confirmation of seroconversion, and false-positive anti-HEV IgM results cannot be entirely excluded, particularly in patients with concurrent viral infections. Additionally, the lack of systematic HEV-RNA testing and genotyping limits virological characterization of the cohort. Although indirect evidence supports genotype 3 involvement, definitive confirmation was not possible.

Finally, host genetic factors were not assessed. While emerging data suggest a role for genetic susceptibility in prolonged cholestatic phenotypes, this aspect could not be evaluated in the present study.

## 5. Conclusions

In this single-center cohort of hospitalized hepatitis E patients from Craiova, Romania (2024–2025), admission laboratory profiles were consistent with acute hepatitis, characterized by marked transaminase elevations and a substantial icteric burden. Across hospitalization, ALT and AST decreased markedly and consistently at the individual level, supporting a robust biochemical recovery signal for hepatocellular injury in most cases. In contrast, bilirubin fractions improved more modestly, and clinically relevant hyperbilirubinemia remained frequent at discharge, underscoring that biochemical resolution of cholestasis may lag behind transaminase normalization in hospitalized hepatitis E.

Sex-stratified analyses suggested a more pronounced cholestatic/icteric phenotype among male patients, evident across continuous bilirubin measures and clinically meaningful bilirubin thresholds. However, these findings should be interpreted cautiously, given the small sample size, retrospective design, and the absence of standardized clinical confounders and mechanistic data. Age-class stratification (≤55 vs. >55 years) did not materially refine admission severity or in-hospital recovery patterns in this small cohort. Larger, multicenter studies with standardized clinical covariates and post-discharge follow-up are warranted to validate these exploratory findings and clarify whether apparent sex-specific differences reflect baseline severity, delayed presentation, host factors, or distinct disease trajectories.

## Figures and Tables

**Table 1:** Baseline characteristics and admission laboratory profile of hospitalized hepatitis E patients.

Variable	Value
Patients, *n*	40
Year (2024/2025), *n*	20/20
Female/Male, *n*	22/18
Age, years	57.5 [38.8–63.0] (16–85)
In-hospital death, *n* (%)	2 (5.0%)
ALT (GPT), U/L	966 [446–1705] (96–3248)
AST (GOT), U/L	800 [352–1231] (181–3764)
Total bilirubin, mg/dL	4.53 [1.60–11.32] (0.72–59.86)
Direct bilirubin, mg/dL	3.04 [1.36–10.28] (0.50–46.80)
Alkaline phosphatase (ALP), U/L	325.50 [240.50–567.50] (78.70–924.00)
GGT, U/L	320.00 [171.25–390.75] (82.00–677.00)
Prothrombin index (IP), %	63.19 [52.17–68.78] (33.00–92.00)
Leukocytes, /µL	7800 [6188–9858] (3650–16.200)
Platelets, /µL	215,000 [198,100–253,750] (63,000–333,000)
De Ritis ratio (AST/ALT)	0.81 [0.59–0.99] (0.38–7.28)

Abbreviations: ALT, alanine aminotransferase; AST, aspartate aminotransferase; ALP, alkaline phosphatase; GGT, gamma-glutamyl transferase; IP, prothrombin index; De Ritis ratio, AST/ALT. Continuous variables are shown as median [IQR] (min–max).

**Table 2:** Paired within-patient changes from admission to discharge in key markers (Wilcoxon signed-rank).

Measure	Admission Median [IQR]	Discharge Median [IQR]	Δ		*p* (Wilcoxon)	*q* (FDR)	Effect r	*n* Nonzero
			Median (ext–int)	95% CI				
ALT (GPT), U/L	966.00 [445.75–1705.25]	432.50 [231.50–634.25]	−372	[−684.50; −270.00]	<0.001	<0.001	−0.642	40
AST (GOT), U/L	799.50 [352.25–1231.00]	190.00 [118.00–311.75]	−527	[−724.50; −214.95]	<0.001	<0.001	−0.740	40
Direct bilirubin, mg/dL	3.04 [1.36–10.28]	2.36 [0.67–7.03]	−1.09	[−1.64; −0.47]	<0.001	<0.001	−0.568	38
Total bilirubin, mg/dL	4.53 [1.60–11.32]	3.27 [1.05–9.00]	−0.95	[−1.78; −0.68]	<0.001	<0.001	−0.578	38
Platelets, /µL	215,000.00 [198,100.00–253,750.00]	243,000.00 [210,500.00–269,000.00]	24,000	[9000; 39,050]	<0.001	<0.001	0.608	40
Prothrombin index (IP), %	63.19 [52.17–68.78]	65.60 [61.25–73.30]	0.92	[−2.09; 5.60]	0.175	0.175	0.220	38
Leukocytes, /µL	7800 [6188–9858]	8715 [7885–9660]	795	[425; 1655]	0.010	0.011	0.408	40
De Ritis ratio (AST/ALT)	0.81 [0.59–0.99]	0.45 [0.32–0.60]	−0.24	[−0.36; −0.21]	<0.001	<0.001	−0.684	40

Δ = discharge − admission; *q*-values represent BH-FDR adjustment across paired endpoints. N = 40 paired observations for all endpoints; ‘n nonzero’ indicates the number of non-zero paired differences (i.e., excluding ties). Effect r denotes the matched-pairs rank–biserial effect size (sign indicates direction of change).

**Table 3:** Sex differences at admission in core laboratory markers (Mann–Whitney U; BH-FDR within this set).

Measure	Female Median [IQR]	Male Median [IQR]	*p* (MWU)	Cliff’s δ	*q* (BH-FDR)
ALT (GPT), U/L	1296.00 [595.25–2213.00]	747.00 [364.00–970.00]	0.045	0.373	0.102
AST (GOT), U/L	1112.00 [307.25–1387.75]	699.00 [620.00–900.00]	0.421	0.151	0.421
Total bilirubin, mg/dL	2.05 [1.53–3.74]	13.10 [8.70–14.77]	<0.0001	−0.737	0.0006
Direct bilirubin, mg/dL	1.80 [1.04–3.08]	11.70 [5.70–14.17]	<0.0001	−0.667	0.001263
ALP, U/L	306.00 [171.50–682.50]	345.00 [248.00–405.00]	0.258	−0.212	0.332
GGT, U/L	366.00 [176.25–452.25]	302.00 [178.00–341.00]	0.178	0.252	0.267
Prothrombin index, %	62.17 [55.55–71.48]	64.21 [41.79–64.21]	0.333	0.181	0.374
Leukocytes, /µL	6230 [5005–8105]	9840 [9070–9920]	<0.001	−0.656	0.001
Platelets, /µL	212,500 [196,700–220,425]	233,000 [214,000–256,000]	0.105	−0.303	0.189

*q*-values are BH-FDR adjusted within this table. Cliff’s δ indicates stochastic superiority (negative values indicate higher values in the male group, given the female–male ordering used here).

**Table 4:** Sex differences at discharge in core laboratory markers (Mann–Whitney U; BH-FDR within this set).

Measure	Female Median [IQR]	Male Median [IQR]	*p* (MWU)	Cliff’s δ	*q* (FDR)
ALT (GPT), U/L	554.00 [194.50–703.50]	276.00 [265.00–488.00]	0.422	0.151	0.422
AST (GOT), U/L	119.00 [70.58–330.00]	193.00 [146.00–298.00]	0.083	−0.323	0.117
Total bilirubin, mg/dL	1.10 [0.80–3.20]	8.30 [7.31–25.76]	<0.001	−0.667	0.001
Direct bilirubin, mg/dL	0.69 [0.49–2.66]	6.70 [5.60–20.39]	<0.001	−0.717	0.0008
Prothrombin index, %	68.05 [65.60–77.51]	62.00 [45.48–63.30]	<0.001	0.595	0.002
Leukocytes, /µL	8010 [6542–8628]	8800 [8800–10,230]	<0.001	−0.616	0.002091
Platelets, /µL	241,000 [216,750–342,250]	245,000 [198,000–251,000]	0.421	0.151	0.422119

*q*-values are BH-FDR adjusted within this table. Cliff’s δ indicates stochastic superiority (negative values indicate higher values in the male group, given the female–male ordering used here).

**Table 5:** Clinically relevant hyperbilirubinemia thresholds by sex (Fisher’s exact).

Endpoint	Female *n*/*N* (%)	Male *n*/*N* (%)	*p*
Admission TB ≥ 2 mg/dL	12/22 (54.5%)	16/18 (88.9%)	0.035
Admission TB ≥ 5 mg/dL	4/22 (18.2%)	14/18 (77.8%)	<0.001
Admission TB ≥ 10 mg/dL	4/22 (18.2%)	12/18 (66.7%)	0.003
Discharge TB ≥ 2 mg/dL	6/22 (27.3%)	16/18 (88.9%)	<0.001
Discharge TB ≥ 5 mg/dL	4/22 (18.2%)	14/18 (77.8%)	<0.001

TB, total bilirubin. *p*-values are from Fisher’s exact test.

**Table 6:** Sex differences in within-hospital change (Δ discharge − admission) (Mann–Whitney U; BH-FDR within this set).

Delta	Female Δ Median [IQR]	Male Δ Median [IQR]	*p* (MWU)	Cliff’s δ	*q* (BH-FDR)
ALT Δ (discharge − admission), U/L	−675.00 [−1526.25–−317.00]	−234.00 [−523.00–24.00]	0.007	−0.494	0.039
AST Δ, U/L	−736.00 [−1269.75–−212.47]	−474.00 [−707.00–−145.00]	0.0341	−0.393	0.069
Total bilirubin Δ, mg/dL	−0.73 [−1.22–−0.27]	−2.61 [−5.90–−0.90]	0.014	0.454	0.039
Direct bilirubin Δ, mg/dL	−0.98 [−1.82–−0.09]	−1.21 [−5.00–−0.10]	0.307	0.191	0.351
Prothrombin index Δ, pp	7.20 [−2.09–13.62]	−0.91 [−5.31–3.00]	0.074	0.333	0.119
Leukocytes Δ, /µL	1440 [590–2428]	390 [−900–1600]	0.105	0.303	0.140
Platelets Δ, /µL	44,000 [13,500–138,500]	9000 [−11,000–21,000]	0.012	0.464	0.039
De Ritis ratio Δ	−0.27 [−0.58–−0.05]	−0.23 [−0.25–−0.12]	0.487	−0.131	0.487

*q*-values are BH-FDR adjusted within this table. Cliff’s δ indicates stochastic superiority (negative values indicate higher values in the male group, given the female–male ordering used here).

**Table 7:** Laboratory profile by age class (≤55 vs. >55 years) (Mann–Whitney U; BH-FDR within this set).

Measure	≤55 Years Median [IQR]	>55 Years Median [IQR]	*p* (MWU)	Cliff’s δ	*q* (BH-FDR)
**Admission**					
ALT (GPT), U/L	1215.00 [962.00–1781.00]	747.00 [351.25–1667.00]	0.178	0.253	0.623
AST (GOT), U/L	900.00 [365.00–1252.00]	699.00 [390.50–1204.50]	0.838	0.040	0.881
Total bilirubin, mg/dL	4.15 [2.05–10.30]	4.91 [1.55–13.92]	0.488	−0.131	0.758
Direct bilirubin, mg/dL	3.43 [1.97–9.80]	2.66 [1.36–11.70]	0.881	−0.030	0.881
Prothrombin index, %	61.53 [45.48–64.21]	64.21 [59.00–78.54]	0.035	−0.394	0.242
Leukocytes, /µL	7190 [4810–9840]	8410 [6270–9900]	0.105	−0.303	0.491
Platelets, /µL	216,000 [196,000–233,000]	214,000 [209,125–255,250]	0.488	−0.131	0.758
**Discharge**					
ALT (GPT), U/L	612.00 [276.00–821.00]	267.00 [170.75–475.50]	0.015	0.455	0.208
Discharge AST (GOT), U/L	235.00 [115.00–353.00]	146.00 [119.00–276.75]	0.488	0.131	0.758
Discharge Total bilirubin, mg/dL	3.82 [0.76–9.30]	2.73 [1.11–8.75]	0.391	−0.162	0.758
Discharge Direct bilirubin, mg/dL	3.27 [0.69–6.90]	1.45 [0.64–7.23]	0.838	0.040	0.881
Discharge Prothrombin index, %	65.60 [63.30–72.40]	65.60 [59.67–74.17]	0.673	0.081	0.881
Discharge Leukocytes, /µL	8630 [6220–10,230]	8800 [8215–9302]	0.360	−0.172	0.758
Discharge Platelets, /µL	245,000 [225,000–256,000]	241,000 [202,000–277,000]	0.754	0.061	0.881

*q*-values are BH-FDR adjusted within this table. Cliff’s δ indicates stochastic superiority (negative values indicate higher values in the male group, given the female–male ordering used here).

**Table 8:** Age-adjusted log-linear models for bilirubin and leukocytes (effect expressed as fold-change).

Time	Model	Term	Effect	95% CI	*p*
Admission	Total bilirubin	male vs. female	4.65×	1.53–14.09	0.015
		per 10-year increase	1.02×	0.79–1.33	0.872
	Direct bilirubin	male vs. female	4.49×	1.29–15.58	0.030
		per 10-year increase	1.01×	0.76–1.36	0.929
	Leukocytes	male vs. female	1.55×	1.12–2.14	0.017
		per 10-year increase	0.99×	0.92–1.07	0.821
Discharge	Total bilirubin	male vs. female	7.10×	2.07–24.40	0.006
		per 10-year increase	0.97×	0.72–1.29	0.812
	Direct bilirubin	male vs. female	8.54×	2.38–30.65	0.004
		per 10-year increase	0.92×	0.68–1.24	0.586
	Total bilirubin + baseline	male vs. female	1.53×	0.78–3.00	0.229
		per 10-year increase	0.94×	0.83–1.08	0.407
	Direct bilirubin + baseline	male vs. female	2.16×	1.08–4.29	0.044
		per 10-year increase	0.91×	0.79–1.04	0.193

Outcomes were modeled on the log scale; effects are exponentiated coefficients (fold-change). The age effect is per 10-year increase.

**Table 9:** Linear models for discharge prothrombin index (effect expressed as percentage-point difference).

Model	Effect of Male Sex (pp)	95% CI	*p*	R^2^
Discharge IP (linear)	−20.75	−36.85–−4.64	0.015	0.303
Discharge IP (linear) + baseline	−13.08	−28.41–2.26	0.089	0.510

pp, percentage points. R^2^ is the model coefficient of determination.

## Data Availability

The data presented in this study are available on request from the corresponding author.

## Abbreviations

The following abbreviations are used in this manuscript:

ALP	Alkaline phosphatase
ALT	Alanine aminotransferase
AST	Aspartate aminotransferase
BH-FDR	Benjamini–Hochberg false discovery rate
CI	Confidence interval
DB	Direct bilirubin
GPT	Glutamate–pyruvate transaminase (ALT)
GOT	Glutamate–oxaloacetate transaminase (AST)
GGT	Gamma-glutamyl transferase
HEV	Hepatitis E virus
IQR	Interquartile range
IP	Prothrombin index
MWU	Mann–Whitney U test
TB	Total bilirubin
Δ	Discharge minus admission (delta change)
